# Serological evidence of hepatitis A, B, and C virus infection in older adults in Khon Kaen, Thailand and the estimated rates of chronic hepatitis B and C virus infection in Thais, 2017

**DOI:** 10.7717/peerj.7492

**Published:** 2019-08-19

**Authors:** Nawarat Posuwan, Viboonsak Vuthitanachot, Teeraporn Chinchai, Rujipat Wasitthankasem, Nasamon Wanlapakorn, Yong Poovorawan

**Affiliations:** 1 Center of Excellence in Clinical Virology Department of Pediatrics, Faculty of Medicine, Chulalongkorn University, Pathum Wan, Bangkok, Thailand; 2 Chumphae Hospital, Chum Phae, Khon Kaen, Thailand; 3 Department of Microbiology, Faculty of Medicine, Srinakharinwirot University, Watthana, Bangkok, Thailand; 4 National Biobank of Thailand, National Science and Technology Development Agency, Thailand Science Park, PathumThani, Thailand; 5 Division of Academic Affairs, Faculty of Medicine, Chulalongkorn University, Pathum Wan, Bangkok, Thailand

**Keywords:** Viral hepatitis, Older adult, Thailand

## Abstract

Hepatitis A (HAV), hepatitis B (HBV), and hepatitis C (HCV) viruses are hepatotropic viruses responsible for acute/chronic hepatitis associated with liver failure, cirrhosis, and hepatocellular carcinoma. Due to the limited data on the prevalence of hepatitis in the older population in Thailand, this study aimed to evaluate the seroprevalence of these viruses in elderly Thais. Using an automated immunoassay, serum samples from individuals older than 60 years of age in Chum Phae district of Khon Kaen province in northeast Thailand were analyzed for anti-HAV (*n* = 93), HBV markers (*n* = 460, HBsAg, anti-HBs, and anti-HBc), and anti-HCV (*n* = 460). Samples were classified into five age groups (61–65, 66–70, 71–75, 76–80, and >80 years). The overall seroprevalence of anti-HAV, HBsAg, anti-HBc, anti-HBs, and anti-HCV was 98.9%, 4.6%, 51.5%, 32.4%, and 1.3%, respectively. When samples were stratified into three groups representing three generations (children/young adults aged 6 months-30 years and middle-aged adults between 31–60 years old from a previous survey, and older adults aged >60 years from the current study), the highest levels of anti-HAV and anti-HBc were found in older adults. Children/young adults had the lowest levels of HBsAg and anti-HCV, and the highest level of anti-HBs. These findings are consistent with the integration of HBV vaccination into the Expanded Program on Immunization (EPI) in 1992 and coincide with increased awareness of blood-borne viral transmission in Thailand. Extrapolating from our data, the estimated numbers of cases of chronic HBV and HCV infection in Thailand in 2017 were 2.2 and 0.79 million, respectively. Thus, effective treatments for viral hepatitis B and C for middle-aged and elderly Thais are needed. This seroprevalence survey could be used to help formulate policies and possible guidelines for treatment and prevention in specific age groups, which is recommended to facilitate the elimination of viral hepatitis by 2030.

## Introduction

Infection by hepatitis A virus (HAV), hepatitis B virus (HBV), and hepatitis C virus (HCV) causes a significant number of viral hepatitis in Asia (WHO, 2017). The major route of HAV transmission is via fecal-oral route, such as drinking water or eating food contaminated with HAV. It can also be transmitted during sexual activity and blood transfusion (Daniels, Grytdal & Wasley, 2009). HAV infection can occur under poor sanitary conditions. Most HAV-infected individuals recover and have life-long HAV antibodies (WHO, 2016). In the 1970s, anti-HAV could be detected in 97% of Thais 16 years of age or older, and seroprevalence in children aged 4.5 years was 50% (Burke et al., 1981). Although the efficacy of the vaccine against HAV infection is 94–100% (Innis et al., 1994; Werzberger, Kuter & Nalin, 1998), HAV vaccination is not included in the national Expanded Program on Immunization (EPI) in many countries including Thailand. Despite this shortcoming, the incidence of HAV infection in Thailand has decreased due to improved hygiene and the availability of HAV vaccination. In 2014, the anti-HAV seroprevalence in Thailand was <20% among individuals aged <30 years old (Sa-Nguanmoo et al., 2016). Furthermore, the age at which 50% of the population possessed ani-HAV has increased to 42 years of age, suggesting that Thailand is transitioning to a very low HAV endemicity.

HBV is transmitted via blood and bodily fluids, therefore HBV carrier mothers can infect their infants via perinatal or horizontal transmission. Since 1992, the World Health Organization (WHO) has recommended that all countries incorporate HBV vaccines in their EPI program (Van Damme, Kane & Meheus, 1997). HBV vaccination among newborns has reduced HBV infection to 1.3% among <5-year-olds worldwide (WHO, 2017). Thailand started a pilot HBV newborn vaccination program in two provinces in 1988 and extended it to 10 more provinces in 1990. Universal HBV vaccination program was implemented in Thailand in 1992 (Poovorawan et al., 2000). Consequently, while 4.5% of individuals born before the HBV integration into the EPI are HBV carriers, those born after had a carrier rate of <1%, of which 0.1% are children aged <5 years (Posuwan et al., 2016). However, the data on HBV infection in the Thai population aged >60 years remain limited.

Liver cancer was the most common cause of cancer mortality in males and the third most common in females in Thailand (Srivatanakul, 2001). A major cause of liver cancer is viral hepatitis, especially HBV and HCV infection (Wanich et al., 2016). Before HCV screening of donated blood, transfusion was a major cause of HCV transmission. Thailand began blood screening in 1991 using a first-generation anti-HCV enzyme immunoassay (EIA), then with a chemiluminescent immunoassay (CLIA) in 1996. From 2006 onward, nucleic acid amplification technology (NAT) was used to screen for HBV DNA and HCV RNA (Chimparlee et al., 2011). With the reduction in the risk of acquiring HCV from donated blood, transmission was still possible from injections caused by unsafe practice by healthcare workers or needle-sharing (Khan et al., 2000). HCV infection can result in chronic liver disease leading to cirrhosis and hepatocellular carcinoma (HCC). There is currently no vaccine to prevent HCV infection, although several direct-acting antivirals (DAAs) are available to treat HCV. They are inexpensive and can clear the virus in 8-12 weeks (WHO, 2017). Presently, the rate of HCV seroprevalence in Thailand has decreased from 2.5% in 2004 to 0.9% in 2014 (Wasitthankasem et al., 2016).

Thais older than 60 years of age are more likely to be infected with these viruses compared to other age groups because they were born prior to widespread knowledge of blood-borne pathogens and are unlikely to have been vaccinated for HAV or HBV. They may have been exposed to unscreened blood products, received organ transplants, shared needles or personal care items such as razors and toothbrushes, or had unprotected sex. As Thailand has an aging population but with limited data on the prevalence of viral hepatitis in older adults, the present study aimed to evaluate the seroprevalence of HAV, HBV, and HCV in Thais. These findings were combined with data from previous studies among individuals ages 6 months to 60 years to obtain a better estimate of the HBV and HCV infection for the entire population.

## Materials and Methods

The study protocol was approved by the Institutional Review Board of the Faculty of Medicine of Chulalongkorn University (IRB number 006/60). The participants were informed of the study objective and written consent was obtained from all participants.

### Population

The sample size in this study was calculated using the Cochran formula (Israel, 1992). With 90% power, the total number of samples required for this study was 384 persons. The number of samples for each age group was extrapolated from the total population for each age group. All participants (*n* = 460) enrolled in this study were adults aged >60 years old. A previous study in Thailand showed that most adults (96.8%) ages >50 years possessed anti-HAV (Sa-Nguanmoo et al., 2016); therefore, at least one-fifth of the samples were needed for testing in this study and we therefore tested 93 randomly selected samples for anti-HAV. Participants were from the Chum Phae district of Khon Kaen province in northeastern Thailand. Several had chronic diabetes mellitus (21.9%), hypertension (43.9%), and heart disease (1.1%). However, individuals with severe chronic illness involving disabilities or being bedridden were excluded from this study.

### Laboratory methods

Serum samples from 460 participants were quantitatively analyzed for HBV markers (HBsAg, anti-HBc, and anti-HBs) and for anti-HCV. Enzyme-linked immunosorbent assays (ELISAs) were conducted using an automated ARCHITECT i1000SR immunoassay analyzer (Abbott, Wiesbaden, Germany). The positivity criteria were based on the manufacturer’s instructions of sample-to-cut off ratio >1 for HBsAg, anti-HBc, anti-HAV, and anti-HC; and ≥1 mIU/ml for anti-HBs seropositivity. Anti-HBs of ≥10 mIU/ml was considered seroprotective.

### Data analysis

Anti-HBs levels were divided into five groups (<1, 1–<10, 10–<100, 100–<1,000 and ≥1,000 mIU/ml), and ≥10 mIU/ml was regarded as seroprotective. However, the geometric mean titer (GMT) was derived from anti-HBs seropositivity defined as ≥1 mIU/ml. The GMT was plotted on a graph with a log_10_ scale. For comparison, a subset of data from Thai individuals aged 6 months to 60 years old living in Khon Kaen province from our previous 2014 national survey (Sa-Nguanmoo et al., 2016; Posuwan et al., 2016; Wasitthankasem et al., 2016) were used, and the total numbers of cases of HBV and HCV infection in Thailand were extrapolated from our current data combined with these previous data. We divided the population into three groups: children/young adults (6 months-30 years; data from the 2014 survey), middle-aged adults (31-60 years old; data from the 2014 survey), and older adults (>60 years old; data from this study). For HBV markers (HBsAg, anti-HBc and anti-HBs) and anti-HCV, we tested 1,063, 570 and 460 individuals, respectively. For anti- HAV, we tested 727, 382 and 93 individuals, respectively. The Thai population census in 2014 and 2017 was obtained from official statistics provided by the Thai Ministry of Interior (Thai Health Promotion Foundation and National Health Security Office, 2011). The chi-square test was used for comparison HBV, HCV infection between middle-aged adults and older adults groups.

## Results

### Number of older adults in the survey and seroprevalence of HAV, HBV, and HCV

**Figure 1:**
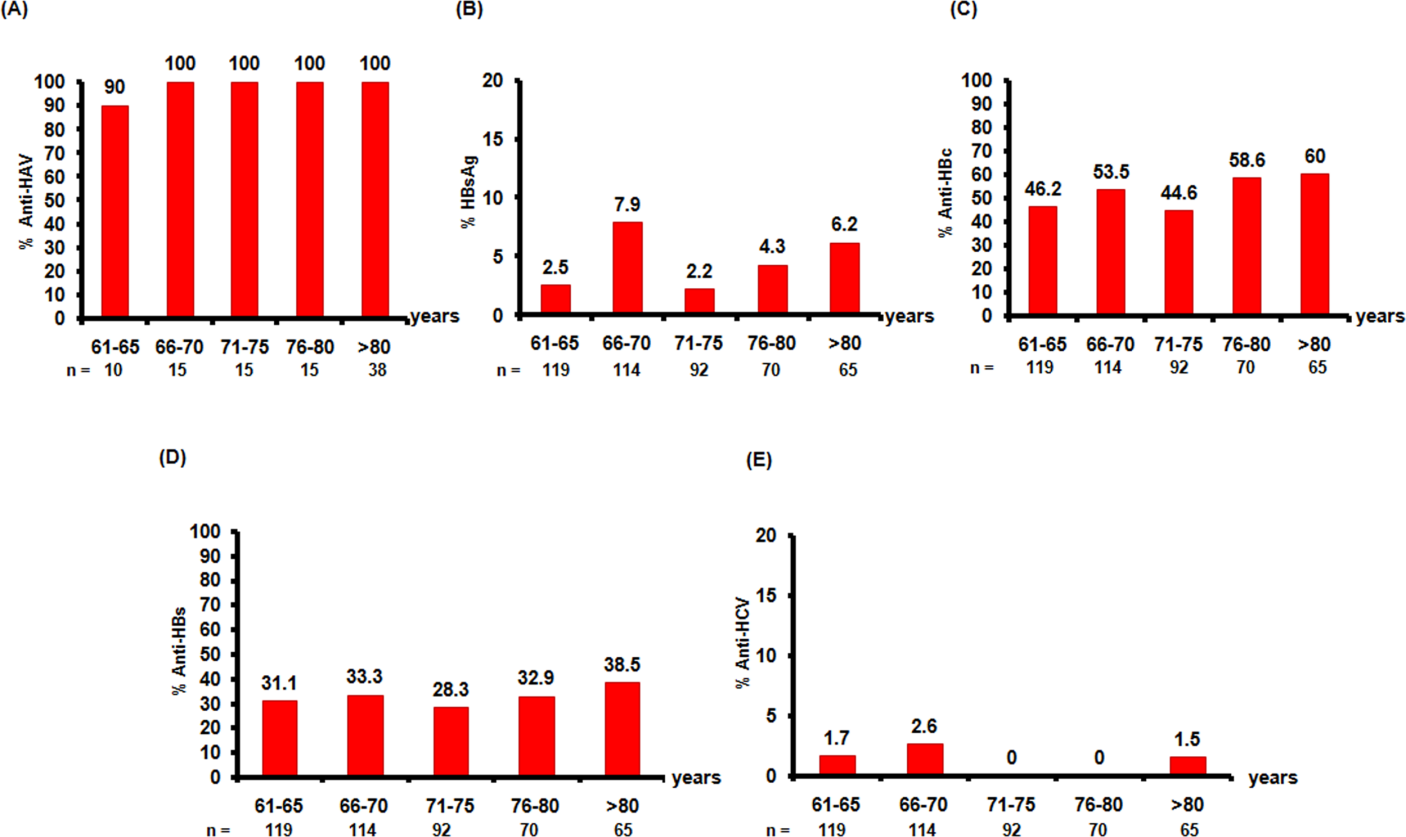
Seroprevalence of anti-HAV, HBsAg, anti-HBs, anti-HBc, and anti-HCV in this study. The five age groups (61–65, 66–70, 71–75, 76–80, and >80 years old) are shown on the *x*-axis. The proportions of individuals positive for (A) anti-HAV, (B) HBsAg, (C) anti-HBc, (D) anti-HBs, and (E) anti-HCV are presented on the *y*-axis. A total of 460 individuals were screened for HBV markers (HBsAg, anti-HBc, and anti-HBs) and for anti-HCV, while 93 individuals were tested for anti-HAV.

Serum samples were collected from 460 healthy older adults who were living in the northeast of Thailand. All samples were tested for three HBV markers (HBsAg, anti-HBc, and anti-HBs) and anti-HCV, while 93 samples were tested for anti-HAV. The number of participants in each age group in the survey is presented in Table 1. Most of the older adults (98.9%) were anti-HAV-positive, while only 1.3% were anti-HCV-positive. Regarding the HBV markers, the seropositivity rates for HBsAg, anti-HBc, and anti-HBs were 4.6%, 51.5%, and 32.4%, respectively (Fig. 1).

**Table 1:** Number of serum samples (stratified into five age groups ) used for determining anti- HAV, HBsAg, anti-HBs, anti-HBc, and anti-HCV levels.

	**HBsAg, anti-HBs, anti-HBc and anti-HCV testing**	**Male**	**Anti-HAV testing**	**Male**
**Age (years)**	**n (%)**	**n (%)**	**n (%)**	**n (%)**
61–65	119 (25.9)	28 (23.5)	10 (10.8)	5 (50)
66–70	114 (24.8)	31 (27.2)	15 (16.1)	8 (53.3)
71–75	92 (20)	27 (29.3)	15 (16.1)	7 (46.7)
76–80	70 (15.2)	27 (38.6)	15 (16.1)	8 (53.3)
>80	65 (14.1)	21 (32.3)	38 (8.3)	14 (36.8)
Total	460	134 (29.1)	93	42 (45.2)

### Seroprevalence of anti-HBs and GMT

**Figure 2:**
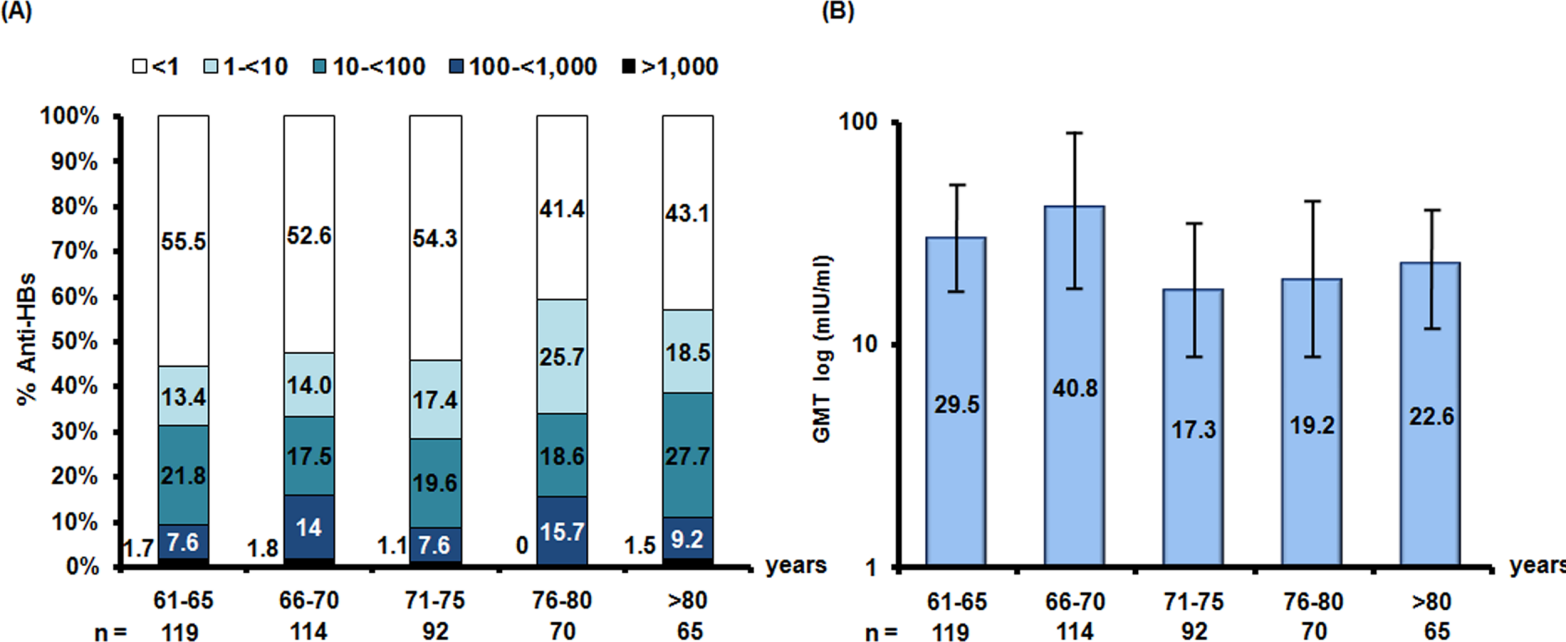
Seroprevalence of anti-HBs and the geometric mean titers (GMTs) in older Thai adults. (A) Anti-HBs levels are indicated as follows: <1 mIU/ml (white), 1 –<10 mIU/ml (light blue), 10 –<100 mIU/ml (blue), 100 –<1,000 mIU/ml (navy), and ≥1,000 mIU/ml (black). Numbers associated with the bar graphs represent percent seroprevalence (*y*-axis). (B) The GMTs derived from anti-HBs ≥1 mIU/ml are in log_10_ scale (*y*-axis). The *x*-axis indicates age groups.

The level of anti-HBs indicates the ability of the immune system to protect against HBV infection; therefore, the concentrations of anti-HBs were divided into five levels (<1, 1–<10, 10–<100, 100–<1,000 and ≥1,000 mIU/ml). Most of the elderly were found to have anti-HBs level <1 mIU/ml. The rate of protective anti-HBs (≥10 mIU/ml) was 32.4%. The GMT was derived from anti-HBs ≥1 mIU/ml. The GMT levels were not much different between age groups, at around 17.3–40.8 mIU/ml (Fig. 2).

### Comparison of HAV, HBV, and HCV seroprevalence among different age groups

**Figure 3:**
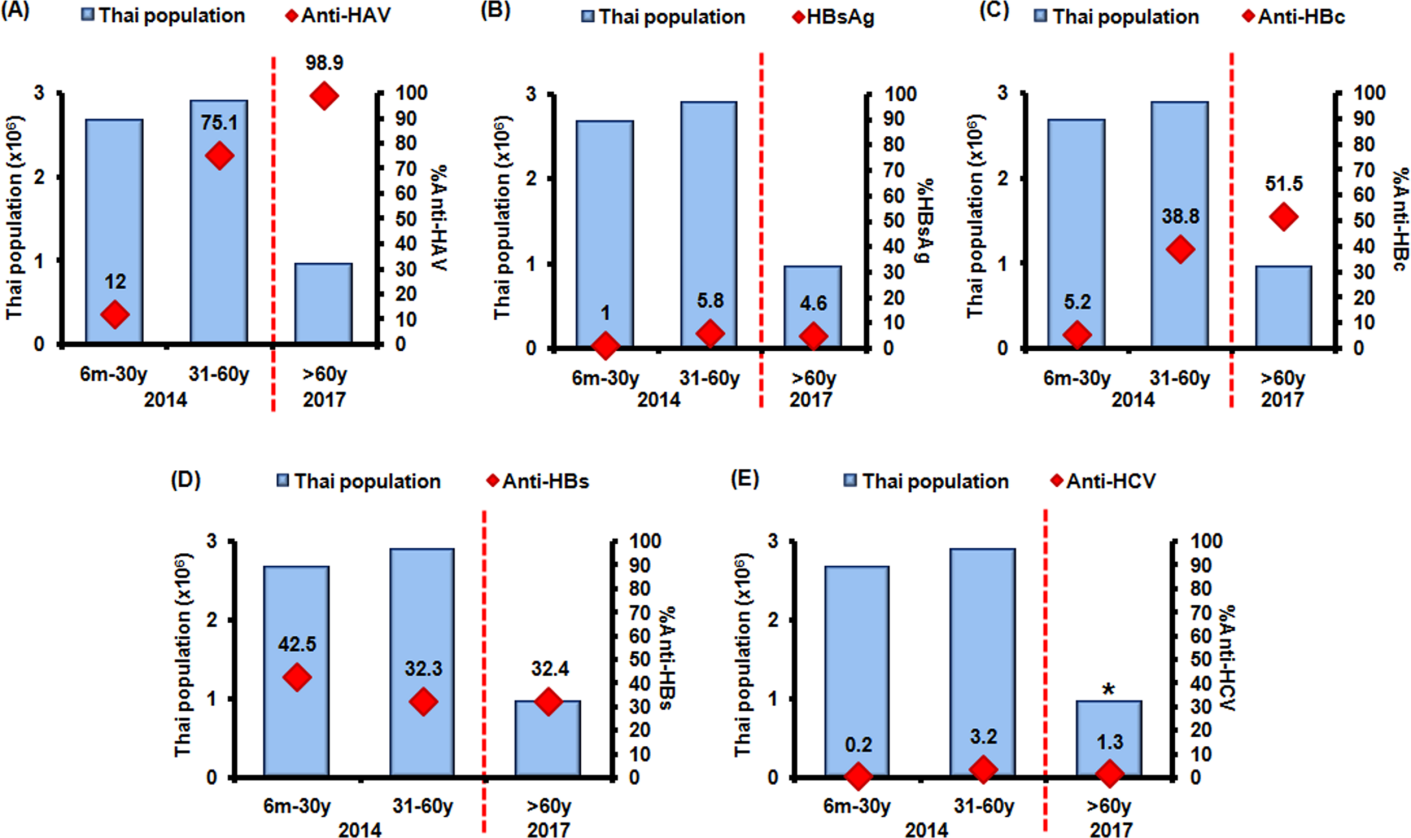
Seroprevalence against HAV (anti-HAV), HBV (HBsAg, anti-HBc, anti-HBs), and HCV (anti-HCV) in different age groups. Thais of different age groups (6 months to 30 years, 31–60 years, and >60 years old, denoted in *x*-axis) were evaluated for (A) anti-HAV, (B) HBsAg, (C) anti-HBc, (D) anti-HBs, and (E) anti-HCV. Population is shown on the *y*-axis. The 2014 study involved age groups 6 month—60 years old, while the 2017 study involved >60 years old. Average serological values are noted (red squares). Asterisk denotes seropositivity rate of statistical significance between middle-aged and older adults (*p* < 0.05).

The seroprevalence of HAV, HBV, and HCV were calculated based on the previous 2014 study data (6 months-60 years old) and data from the present study (>60 years old). The population was divided into three groups, 6 months-30, 31–60, and >60 years old, which represented children/young adults, middle-aged adults, and older adults, respectively (Fig. 3). Most of the older adults had anti-HAV. High levels of chronic HBV and HCV infection were found in middle-aged adults (5.8% and 3.2%, respectively) with lower levels in the older group (4.6% and 1.3%, respectively). The number of HBV carriers among the older age group compared to the middle-aged group was not significantly different (*p* = 0.38). However, HCV serological prevalence was lower in the older age group compared with the middle-aged group (*p* < 0.05).

**Figure 4:**
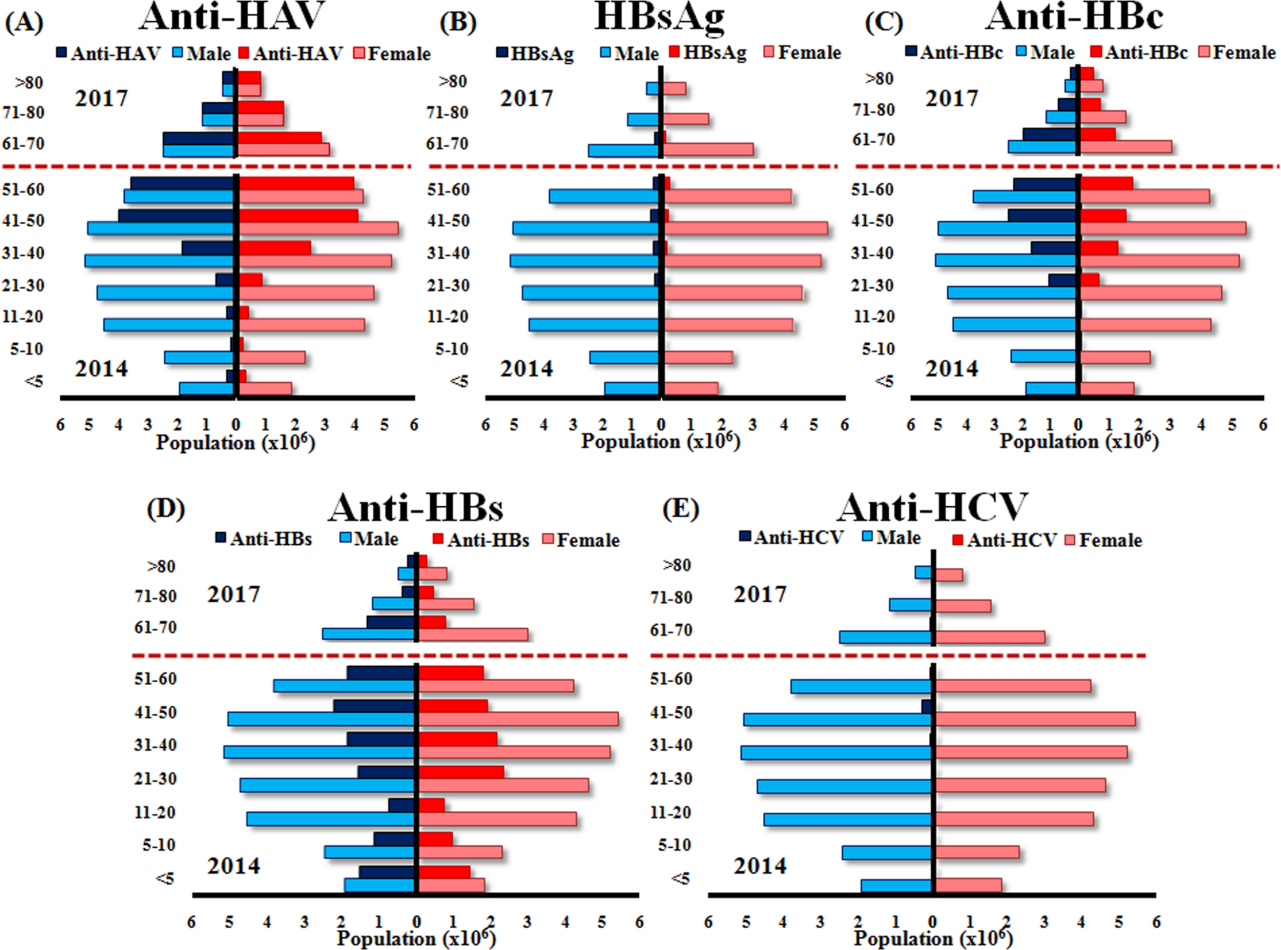
Estimated numbers of males and females positive for anti-HAV, HBsAg, anti-HBc, anti-HBs, and anti-HCV aged <5, 5–10, 11–20, 21–30, 31–40, 41–50, 51–60 (2014 data) and 61–70, 71–80 and >80 (2017 data). The *y*-axis represents the ten age groups in the Thai population. The *x*-axis represents the number (×10^6^) of males and females in Thailand, as indicated by the blue and pink bars, respectively. The navy blue and red bars indicate the number of males and females, respectively, who were positive for anti-HAV (A), HBsAg (B), anti-HBc (C), anti-HBs (D), and anti-HCV (E).

Moreover, population in the northeast of Thailand was divided into ten age groups (<5, 5–10, 11–20, 21–30, 31–40, 41–50, 51–60, 61–70, 71–80 and >80 years old), and the total numbers of males and females and the numbers of males and females who were seropositive for each marker in 2014 and 2017 are shown (Fig. 4). We extrapolated from these data to calculate the total number of chronic HBV and HCV infections in Thailand by using the population level in 2017. Therefore, we estimate that approximately 2.2 and 0.79 million individuals had chronic HBV and HCV infection, respectively (Table 2).

**Table 2:** Number of HBV carriers and anti-HCV-positive individuals (by age group) in the Thai population and extrapolation of the number of cases of chronic HBV infections and anti- HCV-positive individuals in 2017.

**Age (yrs)**	**Population**	**% HBV carrier rate**	Number of HBV carriers^**^	**%HCV anti-HCV +ve**	Number of anti-HCV +ve^**^
<5	3,427,578	0.1	3,559	0.3	10,678
5–10	4,684,121	0.3	13,736	0.4	18,315
11–20	8,315,868	0.7	57,351	0.6	49,158
21–30	9,380,963	3.1	292,719	0.4	41,817
31–40	9,978,123	3.8	376,778	1.0	103,939
41–50	10,433,606	4.7	487,172	2.9	297,716
51–60	8,913,738	6.0	534,112	1.5	130,561
61–70^*^	5,495,458	5.2	283,028	2.1	117,928
71–80^*^	2,706,137	3.1	83,523	0	0
>80^*^	1,291,873	6.2	79,500	1.5	19,875
Total	64,629,482	3.4^***^	2,211,479	1.2^***^	789,987

**Notes.**

* % Carrier of HBV and anti-HCV among age 61–>80 years old were from 2017, while the data on carrier rate of HBV and anti-HCV of age <5–60 year old were from 2014. The Thai population in the year 2017 was used.

** Calculate from % carrier rate in each age group.

*** The total % of carrier rate was calculated from the total number of carrier compare to the total Thai population.

## Discussion

According to the Office of the National, Economic, and Social Development Board of the Prime Minister’s Office, Thailand’s population is rapidly aging. Thailand had 8.4 million older adults in 2010 and is predicted to have around 12.6 million older adults in 2020 (Ministry of Public Health Thailand, Office of the Permanent Secretary, 2017). Healthiness and lifelong immunity against infectious diseases is important for this population group. In all age groups in the study, there were more females than males (though the differences were not significant), possibly because males were less likely to be present during the daytime home visits for blood sampling. However, the female predominance in the older age group correlates with the national data showing higher life expectancy in females compared to males.

A study published 40 years ago found that most Thais were infected with HAV prior to adulthood (Chunsuttiwat et al., 1997). The improvement in sanitation and living conditions, combined with limited HAV exposure among children over time, has contributed to the HAV susceptibility among adults (Sa-Nguanmoo et al., 2016). Studies conducted in developed countries with low rates of HAV infection have reported an increase in morbidity and mortality particularly in the unvaccinated elderly (Ly & Klevens, 2015). Our study revealed that nearly all older adults in the northeast of Thailand (98.9%) had immunity to HAV, which was more than the rates among the children/young adults and middle-aged adults. The immunity to HAV may have been due to natural infection in childhood or during the HAV outbreak periods in 2012 and 2014 in the northeast (Poovorawan et al., 2013a; Sa-Nguanmoo et al., 2016).

The universal HBV vaccination program, which has been integrated into the Thai EPI program since 1992 (Poovorawan et al., 2000), resulted in a decreased seroprevalence of HBsAg (in which HBsAg-positive individuals are potentially infectious carriers) and a decreased seroprevalence of anti-HBc (which indicates current or previous natural HBV infection) (Chongsrisawat et al., 2006). In this study, HBsAg seropositivity was 4.6% in the older group, which was more than in the children/young adult group (1%) (Posuwan et al., 2016). The higher prevalence of chronic infection in the older group and also in the middle-aged adult group (5.8%) may have resulted from being born before HBV vaccination was integrated into the EPI. The anti-HBc seroprevalence due to natural infection was higher in older adults than in children/young adults and middle-aged adults. Thus, the anti-HBc seroprevalence dramatically decreased in people born after the HBV vaccine implementation into the EPI. These results confirm that the rate of natural infection has declined since the integration of HBV vaccination into the EPI.

Interestingly, regarding the rate of anti-HBs ≥10 mIU/ml (which indicates HBV seroprotection), even though the anti-HBs seroprevalence (≥1 mIU/ml) was 32.4% in the older group, only <30% of this group had a seroprotective level of anti-HBs. The rate of anti-HBs (which can occur due to HBV vaccination or a previous natural HBV infection) decreased with time, in the group born after the implementation of the EPI. Even though HBV vaccination has been successfully implemented in many countries for >20 years with good coverage and efficacy among those born after the implementation of the EPI, the rate of anti-HBs seroprotection declined with age and reached its lowest level in individuals aged 11–20 years (Poovorawan et al., 2013b; Posuwan et al., 2018). According to these findings, HBV vaccines should be given to high-risk young adults to boost their immunity and to middle-aged adults who are negative for anti-HBs and anti-HBc to offer seroprotection late in life.

Anti-HCV indicates current or previous HCV infection. The decreasing prevalence of HCV over the past decade may have resulted from several factors (Sunanchaikarn et al., 2007; Wasitthankasem et al., 2016). The discovery of HIV greatly increased public awareness of blood-borne pathogens (Luksamijarulkul et al., 2004). Anti-HCV screening of donated blood was mandated in 1991 and NAT in 2006 to detect viral nucleic acid (Chimparlee et al., 2011). As a result, the number of anti-HCV-positive blood donors slowly declined from 2% in the early year 1990 of screening implementation to 0.5% in 2009 (Chimparlee et al., 2011). The 2014 national anti-HCV surveillance survey found that the seroprevalence was 0.94%; however, among those aged 31–60 years, 0.67% had detectable anti-HCV (Wasitthankasem et al., 2016). As expected, our study found an anti-HCV seroprevalence in older adults of approximately 1.3%, which was lower than the seroprevalence in middle-aged adults (3.2%) in Thailand (Wasitthankasem et al., 2016).

We divided our study population (based on both the 2014 data and the 2017 data from the current study) into three age groups according to major events in society. The population aged <30 years old was born after the EPI implementation and had protection against and awareness of blood-borne transmission. They had a lower seroprevalence of anti-HBc and HBsAg (which occur due to natural HBV infection), a high seroprevalence of anti-HBs (which occur due to vaccination or natural HBV infection), and were nearly all negative for anti-HCV (Posuwan et al., 2016; Wasitthankasem et al., 2016). The middle-aged and older adult groups (aged >31 years) were born before the EPI implementation and were more likely to have experienced intravenous drug use, exposure to non-sterile healthcare practice, and unsafe sexual activity. These two groups had comparably higher seroprevalence rates of anti-HBc, HBsAg, and anti-HCV than the children/young adults group. In contrast, anti-HBs seroprevalence rates were higher in the children/young adults group due to the HBV vaccination.

In the current study, the older group (>60 years) had a slightly lower (but not significantly different) percentage of HBV carriers compared to the middle-aged group. Meanwhile, the anti-HCV seroprevalence was significantly lower than in the middle-aged group. Moreover, HBV and HCV complications (such as cirrhosis and HCC) are more common in late middle-aged to older individuals (Srivatanakul, 2001), which is consistent with data from the nationwide hospital admission data from the Thai National Health Security Office (NHSO) showing that the mean age of patients admitted with cirrhosis and complications was 55 ± 12.8 years (Poovorawan et al., 2015). In central Thailand, the mean age of HCC patients was 57.4 ± 12.7 years (Somboon, Siramolpiwat & Vilaichone, 2014). Tangkijvanich et al. (1999) reported that HCC is more common among men than women, with a peak incidence in the 51–70 age group, and HBV prevalence in HCC patients was 65%, four times the HCV prevalence (17%).

Finally, a molecular epidemiology analysis of HCV genotype 3a in Thailand revealed that this strain spread to Thailand during the mid-1970s and early 1980s. This period overlaps with the Vietnam War (1955–1975) and the period of widespread intravenous drug abuse, thus contributing to the peak incidence of HCV in the middle-aged adults (Akkarathamrongsin et al., 2013). These reasons may explain the observed lower HBV and HCV infection in older adult and the middle-age group in this study.

To extrapolate the potential number of chronic HBV and HCV infection from all age group in Thailand, we combined the data from this study with the previous published report (Posuwan et al., 2016; Wasitthankasem et al., 2016). The results showed that the number of chronic HBV and HCV infections are approximately 2.2 million (3.4%) and 0.79 million (1.2%) individuals, respectively (Table 2). Consequently, Thailand is likely an intermediate chronic HBV and HCV endemic area as a result of the vaccination program and increased awareness of blood-borne infections. It shows that the burden of HBV and HCV in the population remains significant, which will require treatment in order to prevent mortality associated with these viruses.

Our study has several limitations. We only included results from a single province in the northeast of Thailand, which may not be generalizable to the whole country. Furthermore, previous studies have shown that HBV and HCV seroprevalence in the south was lower than the north, northeast and central Thailand (Posuwan et al., 2016; Wasitthankasem et al., 2016). A larger-scale study may reveal differences in the seroprevalence of antibodies related to viral hepatitis in different regions of Thailand. Moreover, it would have been ideal to have evaluated all samples for anti-HAV.

This study showed that although anti-HCV seroprevalence was low among older adults (aged > 60 years), high seroprevalence was observed for anti-HBc, anti-HBs, HBsAg, and anti-HAV among older adults possibly because of poor hygiene, lack of vaccination, and lack of knowledge about blood-borne infections at the time. To prepare to address the needs of the rapidly aging society in Thailand, this seroprevalence survey will be useful in formulating policy and possible recommendations for treatment and prevention guidelines to further reduce HBV and HCV infection-related morbidity and mortality.

##  Supplemental Information

10.7717/peerj.7492/supp-1 Raw Data S1 The participant characteristic and the detection level of anti-HAV, HBsAg, anti-HBs, anti-HBc, and anti-HCV

10.7717/peerj.7492/supp-2 Supplemental Information 1 Case record form

## Additional Information and Declarations

### Data Availability

The following information was supplied regarding data availability: The raw data is available in the Supplemental Files. The raw data shows the serological of anti-HAV, anti-HBs, anti-HBc, HBsAg and anti-HCV in Thai older adults (>60 year old).
