# Supplementary material for: Serological evidence of hepatitis A, B, and C virus infection in older adults in Khon Kaen, Thailand and the estimated rates of chronic hepatitis B and C virus infection in Thais, 2017
Source: PeerJ. 2019 Aug 19;7:e7492. doi: 10.7717/peerj.7492 (PMC6705384; doi:10.7717/peerj.7492)
Supplement: Supplemental Information 1 [file peerj-07-7492-s002.doc]

Firstname……………………………………………Last name………………………………………..

Gender ⬜ Male ⬜ Female Age……………..year Date of birth..…DD...…**/**…MM.…**/**…YY…

Have you ever received Hepatitis A vaccine ⬜ No ⬜ Yes

Have you ever received Hepatitis B vaccine ⬜ No ⬜ Yes

Do you have the chronic disease such as

Chronic hepatitis ⬜ No ⬜ Yes

Diabetes mellitus ⬜ No ⬜ Yes

Hypertensive heart disease ⬜ No ⬜ Yes

Heart disease ⬜ No ⬜ Yes

*** This information will be keptanonymous and use for research only***
